# The Cellular Bases of Antibody Responses during Dengue Virus Infection

**DOI:** 10.3389/fimmu.2016.00218

**Published:** 2016-06-06

**Authors:** Juan Carlos Yam-Puc, Leticia Cedillo-Barrón, Elsa Maribel Aguilar-Medina, Rosalío Ramos-Payán, Alejandro Escobar-Gutiérrez, Leopoldo Flores-Romo

**Affiliations:** ^1^Department of Cell Biology, Center for Advanced Research, The National Polytechnic Institute, Cinvestav-IPN, Mexico City, Mexico; ^2^Department of Molecular Biomedicine, Center for Advanced Research, The National Polytechnic Institute, Cinvestav-IPN, Mexico City, Mexico; ^3^Faculty of Biological and Chemical Sciences, Autonomous University of Sinaloa (UAS), Culiacan, Sinaloa, Mexico; ^4^Department for Immunological Investigations, Institute for Epidemiological Diagnosis and Reference, Health Secretariat, Mexico City, Mexico

**Keywords:** dengue virus, *in vivo* B cell responses, plasma cells, memory B cells, antibodies

## Abstract

Dengue virus (DENV) is one of the most significant human viral pathogens transmitted by mosquitoes and can cause from an asymptomatic disease to mild undifferentiated fever, classical dengue, and severe dengue. Neutralizing memory antibody (Ab) responses are one of the most important mechanisms that counteract reinfections and are therefore the main aim of vaccination. However, it has also been proposed that in dengue, some of these class-switched (IgG) memory Abs might worsen the disease. Although these memory Abs derive from B cells by T-cell-dependent processes, we know rather little about the (acute, chronic, or memory) B cell responses and the complex cellular mechanisms generating these Abs during DENV infections. This review aims to provide an updated and comprehensive perspective of the B cell responses during DENV infection, starting since the very early events such as the cutaneous DENV entrance and the arrival into draining lymph nodes, to the putative B cell activation, proliferation, and germinal centers (GCs) formation (the source of affinity-matured class-switched memory Abs), till the outcome of GC reactions such as the generation of plasmablasts, Ab-secreting plasma cells, and memory B cells. We discuss topics very poorly explored such as the possibility of B cell infection by DENV or even activation-induced B cell death. The current information about the nature of the Ab responses to DENV is also illustrated.

## Introduction

Dengue virus (DENV) is one of the most significant human viral pathogens transmitted by mosquitoes and causes every year ~390 million infections worldwide, resulting in around 500,000 people with severe dengue (SD). It is estimated that over 50% of the world’s population is now at risk of dengue infection, caused by four serotypes (DENV1–4), which circulate in tropical and subtropical regions (1). It is believed that the vast majority of dengue infections are asymptomatic; however, a proportion manifests as a non-specific febrile illness or progresses to classical dengue fever (DF), characterized by fever and severe joint pain. Some of those infections can evolve to SD, such as dengue hemorrhagic fever (DHF) or dengue shock syndrome (DSS) (1). Neutralizing memory antibody (Ab) response is one of the most important mechanisms to defeat both homotypic and heterotypic reinfections with DENV and is therefore the aim of vaccines (2–5). However, one of the main hypotheses about SD revolves around class-switched memory Abs, in a mechanism referred to as Ab-dependent enhancement (ADE) of the infection (6). Although this mechanism has been studied *in vitro*, its potential importance *in vivo* is only beginning to be elucidated (7, 8). Classical epidemiological studies indicate that individuals having a secondary infection with a DENV serotype different to the first one are at increased risk of developing SD (9–11). This includes circumstances such as infants infected for the first time but who already bear maternally acquired DENV-specific Abs (12), which would predispose them to SD. While submitting this review, a report linked Zika virus infection with Guillain–Barré syndrome (13). Of note, there was concomitance of Zika infection, Guillain–Barré syndrome, and the presence of anti-DENV IgG Abs too, suggesting a relationship among these events. At least three preliminary scenarios are envisaged: (a) cross-reactive memory anti-DENV response may contribute to the Guillain–Barré syndrome (apparently discarded in the study), (b) anamnestic anti-dengue IgG responses might have been boosted by Zika in the Guillain–Barré syndrome, or (c) Zika induced cross-reactive Abs to DENV (13, 14). Of note, this is still preliminary and rather speculative, and more solid evidence is needed. What is clear, however, is that the involvement of Ab responses needs very careful scrutiny, and this recent finding highlights the importance of studying the B cell responses not only in DENV but also in these other emerging flaviviruses infections. It is conceivable that memory responses to DENV could be involved in these other flaviviruses diseases.

**Figure 1:**
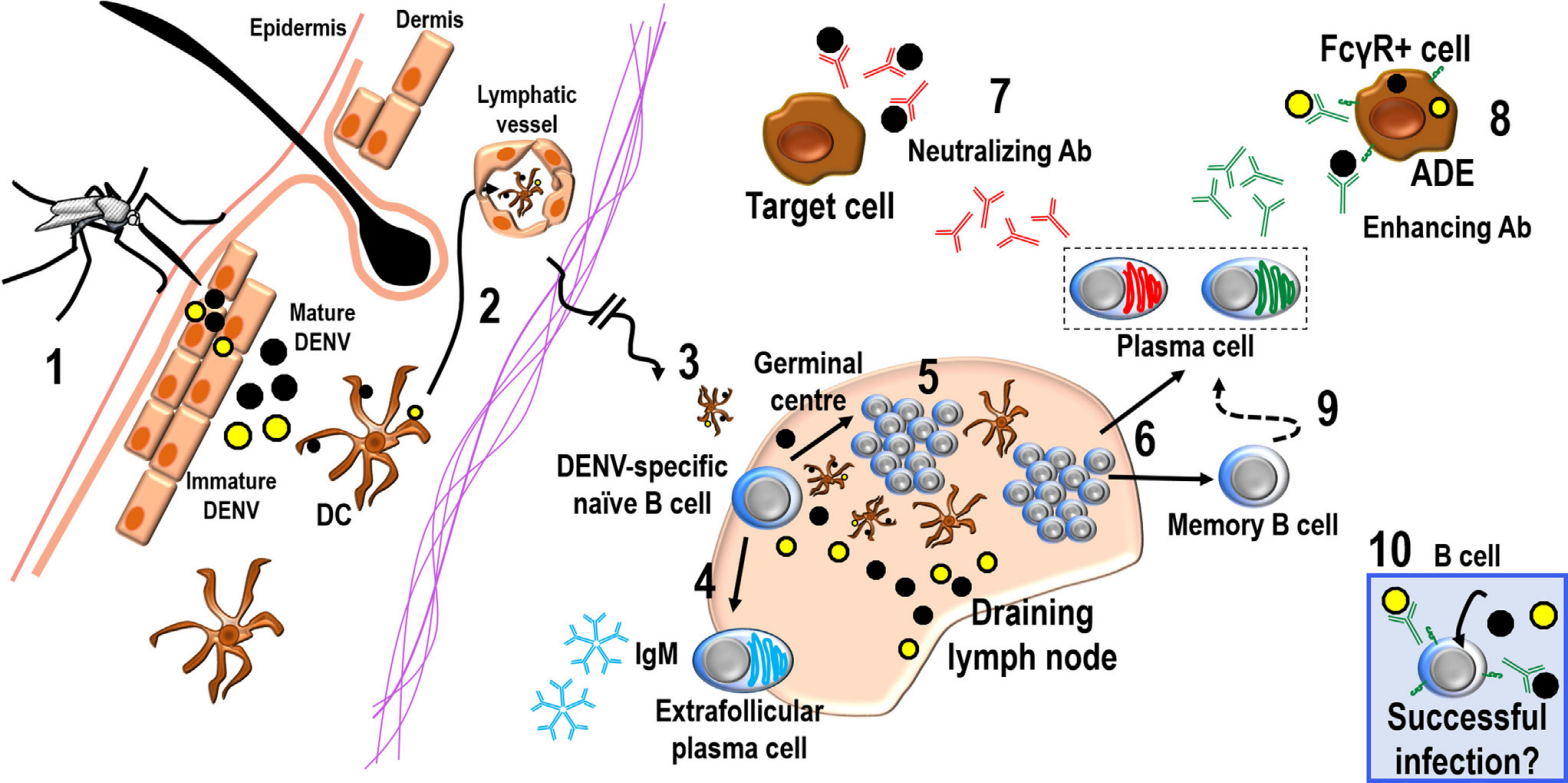
**The B cell responses during DENV infection**. Mosquitoes inoculate DENV mostly intradermally (1); inoculum is a mixture of mature (black circles) and immature (yellow circles) virions. DCs would capture DENV or DENV Ags and enter lymphatics (2) ferrying these Ags to regional DLNs (3). On the other hand, DENV could also reach the DLN *via* the lymph flow in a putative cell-free manner. Upon arrival into DLNs, viruses can encounter DENV-specific naive B cells and could generate short-lived PCs producing IgMs by a T-cell-independent extrafollicular B cell response (4) or could enter to a T-cell-dependent GC reaction (5). GCs will generate long-lived PCs and MBCs (6), which can produce a mixture of both neutralizing and cross-reactive DENV-specific Abs. These Abs would either neutralize the virus, containing the spread of infection (7) or enhance the infection of other targets cells, according to ADE (8). Cross-reactive non-neutralizing Abs seem to predominate in the memory response by MBCs (9). On the other hand, DENV may infect B cells “directly” either in circulation or in tissues such as in secondary lymphoid organs (10).

While T cell responses during acute DENV infection have been studied in some detail, much less is known about the complex mechanisms of B cell responses. Despite that memory Abs are generated by B cells, and that several recent elegant studies are still defining crucial features about the Abs to DENV [for instance, the antigenic epitopes that induce either neutralizing or non-neutralizing Abs (7, 8, 15)], we know surprisingly little about the B cell response itself, either during acute infection when disease is still manifested or regarding the mechanisms generating long-lived plasma cells (LLPCs) or memory B cells (MBCs). Herein, we provide an updated view of the immune response to DENV infection from the B cell perspective: since the early viral entrance into regional lymph nodes (LN) after cutaneous infection, highlighting B cell activation and proliferation or activation-induced B cell death, to the induction of germinal center (GC) B cells, plasmablasts (PBs), plasma cells (PCs), and MBCs, we also illustrate some current information about the cellular bases of the Ab response to DENV antigens (Ag) (Figure 1).

## Dengue Virus

Dengue virus is an enveloped plus strand RNA virus whose genome encodes three structural proteins – capsid, envelope (E), and membrane (M) – and seven non-structural (NS) proteins – NS1, NS2A, NS2B, NS3, NS4A, NS4B, and NS5 (16, 17). Because M protein is first formed as a precursor called precursor M protein (prM), the maturation process of DENV is directed by the proteolytic cleavage of the prM, producing then totally mature infectious particles (18–20). However, this mechanism is not completely efficient, and fully immature or partially mature virions are produced by host cells. Immature status of virions depends on the prM cleavage, modifying size, and morphology of the particles (21). It is estimated that at least 30–40% of DENV particles released from infected mosquito cells are immature, containing different quantities of prM (22). Thus, in the first instance after viral entrance into the host, the immune system might recognize E, M, and prM Ags from DENV. It has been suggested that in the presence of non-neutralizing class-switched memory anti-prM Abs, even immature and non-infectious virus can enter to the cells *via* Fc gamma receptors (FcγR) and replicate efficiently, leading to more infected cells, potentially contributing to a more severe disease (23, 24). On the other hand, the structural protein E has three domains (EDI-III) (25), and it is known that EDIII is involved in the virus attachment to host cell surface (26). Also, it has been known that neutralizing Abs are preferentially directed to EDIII; however, recent findings indicate that Abs to E protein might facilitate DENV infection when present at subneutralizing concentrations (7, 15). Likewise, it has been also proposed that Abs to the whole protein E can also behave as facilitating ones by enhancing infectivity of immature or partially mature particles due to recognition of epitopes that are exposed in immature virions (27, 28). In addition to the potential facilitating effects of these Abs, those that are indeed neutralizers seem to be directed against complex conformational epitopes, which are expressed only when proteins are already assembled on a mature virus particle; therefore, it has been complicated to dissect the precise antigenic nature of these structures (2). Nonetheless, the currently preferred animal model to study the *in vivo* immune response to DENV infection is mostly limited to immune-deficient mice [a mouse deficient for both α/β and γ interferon receptors in a 129 background (AG129)] (29–31). However, to assess precisely the intact immune responses to DENV and – for instance – the generation of potentially neutralizing or non-neutralizing Abs to this virus, these animals might not be the best indicated model.

In addition to this, despite the fact that only the E protein is exposed on the surface of the fully mature virions, the antigenic structure of DENV is very complex, since there are conformational changes in DENV morphology along the replication cycle, such as the different structures found in mosquitoes and humans. Thus, the ideal DENV vaccine candidates should generate an optimal humoral response with Abs that bind to and neutralize the whole spectrum of viral structures (32).

## B Cell Infection and Activation by DENV

For many years, monocytes were deemed the main primary target cells for DENV (33–35). However, the precise mechanisms of *in vivo* and *in vitro* infection of these cells are still controversial, and the percentage of circulating DENV-infected monocytes is too low (36). It could be that after activation, monocytes may get infected, since in circulation, they are mostly resting or immature (37, 38), thus somehow “resistant” or not permissive to the infection. Many reports have used these cells to assess ADE *in vitro* due to their FcγRs, obtaining an increased frequency of infection (34); nevertheless, the proportion of infected monocytes is still low. Being B cells, the ones responsible for the Ab responses, not much is known about their role during DENV infection or whether they could be themselves targets of DENV. Studies *in vitro* have shown that human lymphoblastoid cell lines with B cell characteristics were productively infected by DENV2 (39). Also, although many efforts have been done to elucidate the fraction of peripheral blood mononuclear cell (PBMC) infected with DENV during acute disease, there are no definite evidences yet and little is known about the cell types infected *in vivo*. There are reports that B cells are themselves targets of DENV infection. In particular, one study has identified B cells as the major DENV-infected cell population from the PBMCs. Cells were collected from acutely ill dengue patients and separated into subsets. The majority of the virus was recovered from the B cell subset, and this was irrespective of the DENV serotype. Interestingly, DENV was not recovered from monocytes or NK cells (40). It has also been shown that CD19+ cells increase during DENV infection and that these increments correlated with the presence of so-called atypical lymphocytes seen in Giemsa-stained blood films. These atypical lymphocytes were defined as large mononuclear cells having a fine homogeneous nuclear chromatin and a dark staining cytoplasm, some of these cells resembled blast cells. In addition, these atypical lymphocytes accounted for 10% or more of PBMCs in patients with DF or DHF (41). However, the origin of these atypical lymphocytes is still unclear, and B cells have been suggested as the source due to the increase of CD19+ cells in DHF patients (41, 42). It is possible that this population of atypical lymphocytes corresponds to PBs, since PBs responses seem to dominate the B cell compartment during DENV infection, as discussed below.

In another study (43), it was shown that B cells are the predominant DENV-infected cells in dengue patients, with 20–81% of CD19+ cells in the PBMCs containing DENV3. Nevertheless, other *in vitro* studies have found different results. For instance, human splenic macrophages, but neither T nor B cells, appeared to be permissive for DENV infection, and these splenic macrophages exhibited enhanced DENV infection in the presence of highly diluted DENV-immune human serum (44).

In a report analyzing one patient with DHF, it was possible to isolate two different genotypes of DENV2, and high levels of viral Ags were detected in peripheral B lymphocytes; one of the isolated viruses was capable of infecting and effectively multiplying in a B cell line. However, the other isolated virus did neither efficiently bind to nor was able to infect the B-cell line (45). It could be that the infection of B cells during dengue is dependent on the DENV genotype or serotype. Also, it is important to note that B cells might get infected *via* FcγRs if Abs indeed facilitate the invasion of host cells during secondary heterologous infections. In fact, from this perspective alone, not only B cells but also any cell bearing FcγRs is potentially susceptible of DENV infection.

Are B cells infected with DENV? Are they infected only in peripheral blood or in tissues too? There are few reports suggesting DENV infected B cells in tissues. Describing the distribution of the DENV-Ags in fatal cases in humans, the presence of viral Ags was found inside lymphoid organs. Positivity for DENV proteins has been found in blast cells inside B cell follicles, PCs, and B cells in spleen and LNs (46). The presence of positive-strand viral RNA has been related to viral replication in GC B cells from humans (47) (GCs will be described below). On the other hand, NS3, NS1, prM, and E viral proteins have been reported inside GCs in LNs from both humans and mice (48–50), suggesting infection of GC B cells. These findings showed DENV Ags inside lymphoid tissues, particularly inside GCs, suggesting a potential infection of B cells in these structures. Perhaps B cells during GC reactions express molecules that DENV can target as receptors to infect them. Nevertheless, it is feasible that DENV Ags reach B cell follicles by other routes (such as lymph, blood, or the complex intranodal conduit system), and the presence of DENV Ags inside B cell follicles does not necessarily indicate infection of B cells.

Altogether, these data suggest that B cells could indeed get infected by DENV, but are they activated by the virus or by other cells in the microenvironment during infection?

Analyzing the gene expression in B cells during their interaction with DENV *in vitro*, it was described that in response to this virus, B cells over-expressed several genes such as TRAIL, IP-10, and MCP-2 (51). Another report assessed the permissiveness of human B cells to DENV2 infection indicating active DENV2 replication, also that the infection induced IL-6 and TNF-α production by B cells. Furthermore, heterologous serum from patients infected with DENV3 was able to increase the proportion of DENV2-infected B cells and the cytokine production by these B cells (52).

During DENV infection, the interaction between infected and non-infected cells and the release of inflammatory mediators may play an important role in the outcome of the disease. Some reports have described the activation of B cells by other DENV-infected cells. For instance, it was described that murine splenic B cells can be efficiently activated *in vitro* and *in vivo* by DENV-infected peritoneal macrophages, leading to the clonal expansion of those B cells, as authors showed by counting the virus-specific IgM Ab plaque-forming cells (53). On the other hand, the help of T cells by direct cell contact, and the cytokines from macrophages were necessary, *in vitro*, for activation of splenic B cells (54). A/J inbred mice that were inoculated intravenously with a non-mouse-adapted DENV2 showed early activation of B lymphocytes and IgM production. These IgM-producing B cells may be important for clearing primary DENV infection (55), but more studies on this are needed.

In sum, many reports suggest that B cells are themselves infected by DENV, but this infection could depend on the serotype or genotype of the virus, as many other features of the infection, such as, for instance, the expression of FcγRs and the history of previous DENV infections. Moreover, it could be that either DENV or other infected-target cells, such as macrophages, might activate B cells that can then contribute to the release of inflammatory mediators. Thus, in addition to producing Abs, B cells may be playing an important role in activating the immune system or in inflammatory reactions during acute DENV infection.

## Plasmablasts and Plasma Cells Responses during DENV Infection

Classical epidemiological studies indicate that SD is more common in secondary heterologous DENV infections (9–11), implying the involvement of immune mechanisms. Efforts to understand the immune bases of this SD have correlated cross-reactive class-switched (memory) non-neutralizing Abs from a previous infection with the enhancing effect upon new DENV-infected cells, among other mechanisms. These memory IgG Abs would be serotype cross-reactive and non-neutralizing. Since Abs derive from B cells, this implies that the mechanisms of B cell activation, maturation, and differentiation are important in determining the subsequent clinical outcome of the disease. Of note, these memory responses are dependent on T-cell help. The study of the basic cellular mechanisms for Ab production has been rather neglected in dengue. For instance, it was not until very recently that the potential role of PBs and PCs during DENV infection was evaluated, even when these latter cells are the actual Ab-producing cells. PBs responses in patients with acute DENV infection show a big increase over the levels observed in patients with other viral infections, such as influenza, and over the baseline levels found in non-infected healthy subjects (56–60). The global gene expression patterns in PBMCs isolated from DHF patients showed an enrichment of PBs signatures that was accompanied by an increase of PBs by FACS analysis (60). Of note, the amount of PBs is higher even in DENV-infected children than in control healthy subjects (59). As high numbers of PBs and PCs have been observed in dengue patients, this suggests increased Ab production. The numbers of PBs and PCs were higher in secondary than in primary dengue cases, suggesting re-activation of MBCs. In addition, DENV seems to activate polyclonal B cells that cross-react with other Ags, such as polio virus, since significant amounts of polio-reactive Abs were identified 15–25 days after fever in dengue patients compared to control subjects (56).

It is worth mentioning that PBs responses seem to overtly dominate the B cell compartment (often as much as 80% of the CD19+ cell population were PBs) during DENV infection, making up as much as 30% of the total peripheral lymphocytes (58). In contrast, influenza vaccine or primary vaccination with the yellow fever vaccine induced a much smaller PBs response, around 2–3% of the total B (CD19+) cells (61). The PBs during DENV infection are present in the peripheral circulation only for a relatively short time, undergoing contraction or migration to tissues where long-term Ab production can be sustained. However, only a small number of them survive long-term as LLPCs. It could be that the majority of the induced PBs is predestined to a short life span (58). The authors suggested that DENV could be inducing the surviving of cross-reactive B cells, since the increase in poly-serotype-specific PCs during a secondary DENV infection seem to be mediated by the cross-reactive MBCs formed during prior heterologous infections (59, 62).

Although in some cases PBs show a strong polyclonal response to the E protein, their specificity is not representative of the serum Abs secreted by LLPCs in the memory phase (63). In search of a potential dengue-specific genetic pattern regarding the usage of V and J genes for both H and L chain, it was described in DENV-specific and non-specific PBs isolated from secondary infections, that DENV-specific PBs showed a preference for VH1 family, whereas VH3 gene usage was dominant in MBCs (63). Authors suggested that DENV might selectively bind to B cells using rather unusual V family genes and these B cells would be efficiently activated and differentiate into PBs during acute disease. Compared with the pauci-clonal response seen to influenza vaccines in subjects with pre-existing immunity, PBs responses to DENV infection were relatively polyclonal (61, 63).

Considering the neutralizing Ab responses to DENV, the data suggest that after secondary infections, neutralizing Abs are produced by newly activated B cells. This is because there is an increased response by cross-reactive MBCs producing cross-reactive and non-neutralizing Abs, which would rather enhance the infection instead of controlling it (56). According to this, the neutralizing Abs would be produced by recently activated B cells. Understanding the nature of activated or re-activated B cells is of particular relevance in the context of efficient and safer human vaccination efforts. It will be important to determine the extent to which defined Ag-specific PBs clones are selected from the memory pool and whether they modify their specificity or affinity in the memory phase, as this could help to determine more accurate correlates of protection (63). It is unknown whether a secondary encounter during a heterologous DENV infection will modify the affinities from the cross-reactive MBCs generated during the first GC reactions.

Additionally to an association of high PBs numbers with severe secondary DENV infection and with the production of memory Abs that cross-react with heterotypic DENV serotypes, DENV also induces B cell activation, proliferation, and cell death, mostly in patients with SD, suggesting that DENV infection promotes activation-induced B cell death and perhaps increased B cell turnover. Cell death has been determined by the expression of caspase-3, a marker of apoptosis, and by the increased expression of the pro-apoptotic marker CD95 (57). Analyzing the apoptotic genes upregulated in PBMCs during the acute phase of natural DENV infection, in patients with SD, it was found the upregulation of the B cell translocation gene 1 (*BTG1*), and of many other apoptotic genes in B cells. However, it was not analyzed which PBMCs subsets underwent apoptosis (64). Another report showed that an apoptotic CD8+ T cell population is increased among the apoptotic PBMCs in patients with SD, but apoptosis in the B cell fraction was not examined (65). Is DENV inducing death in B cells? Is apoptosis the mechanism for this cell demise? Of note, apoptosis is a “silent” form of death that prevents, rather than promotes, inflammation. Is this activation-induced B cell death an advantageous mechanism for the virus to successfully establish the infection or to avoid overt inflammation by inducing apoptosis?

The gene expression pattern in the PBs fraction of PBMCs from DHF patients showed that the cell cycle/endoplasmic reticulum gene cluster displayed a strong positive correlation with CD19+ lymphocytes. Flow cytometry analysis revealed that most PBs/PCs also expressed the cell cycle-associated nuclear Ag Ki67, indicating that they were indeed proliferating (60). Genes associated with the regulation of apoptosis were also found among the group whose transcripts were more abundant during early DENV infection, which correlates with the expression of apoptosis markers such as caspase-3 and Fas in PBs and B cells (57, 60). Altogether, the data suggest that DENV induces a strong B cell response dominated by cross-reactive PBs during the acute phase of the infection; this response includes cell proliferation and cell death, apparently by non-inflammatory apoptosis, and also an increased B cell turnover.

It is important to highlight that, despite the difficulties, the majority of reports on B cell responses during DENV infection is based on human samples. Trying to overcome some of these complications, other studies with DENV have used animal models, for instance, mice; however, very few findings have been reported regarding the B cell responses. In one of them, DENV-infected mice were challenged with LPS, and the Ab response against this molecule was evaluated. The response to LPS was significantly lower in DENV-infected mice in comparison with animals inoculated with control conditions such as inactivated DENV. These results suggest that DENV infection in mice may decrease intrinsic B-cell functions and that this immune “suppression” might be caused by active viral replication and not by the viral Ags themselves, since the administration of inactivated DENV failed to cause this decreased immune response (66).

Another study showed that after secondary DENV infections in the immune-deficient murine model (AG129 mice), increased DENV-specific avidity in Abs was not associated with increased DENV-specific neutralizing Abs, whose production appears to be mediated by naive B cells (67).

It is important to emphasize that despite the fact that class-switched PBs, PCs, and MBCs are generated and selected mostly in GC reactions, so far, there are no studies about the potential involvement (or not) of GCs during DENV infection. The GC is a very complex microenvironment, where clonal B cell expansion and selection occurs in response to T-cell-dependent Ags. Two crucial molecular mechanisms are utilized in the GCs, somatic hypermutation and class-switch recombination. The outcome of the GC reaction is the generation of long-lived, high-affinity Ab-secreting cells/PCs, and MBCs, thus developing both immediate and long-term protection against re-infections (68–70). However, we do not know how the affinity is modified, especially during secondary homologous and heterologous DENV infections. Our group has recently shown that cutaneously delivered DENV has the ability to infect immune-competent mice inducing a strong GC response. The overall outcome of these GC responses seems a bigger quantity of prM-specific GC B cells and a higher titer of Abs to prM than those to the E viral protein (50). It is conceivable that DENV proteins might be inducing both neutralizing and non-neutralizing memory Abs; some of these (the non-neutralizing ones) would potentially enhance the infection of other target cells *via* FcγRs, according to the ADE hypothesis, thus ensuring successful secondary heterologous infections. Moreover, it is unknown whether cross-reactive memory Abs from the first DENV encounter modify the activation, affinity maturation, and selection of B cells during GC reactions in heterologous subsequent DENV infections.

## Primary B Cell Responses and IgM Abs during DENV Infection

On first encounters with hosts, most pathogens will elicit a primary humoral immune response characterized by an early rise of Ag-specific IgM Abs in a T-cell-independent extrafollicular reaction, and if complex circumstances allow it, this might be followed by affinity maturation, isotype Ab switching, and the ensuing increase of Ag-specific IgG, IgA, or IgE Ab titers, but now in a T-cell-dependent manner (71). For instance, primary IgM Abs provide protection, and their absence in influenza-infected mice triggered increased viral loads in the lungs, with significantly reduced levels of virus-specific IgG1 and IgG2a Abs (72). Of note, despite the fact that DENV-specific IgM has long been used as diagnosis for DENV infection (73), the precise B cell source and the putative effector mechanisms of these IgM Abs are unclear. IgM Abs could come from several possible B cell sources such as from extrafollicular reactions by B1 B cells, from transitional B cells, or from marginal zone (MZ) B cells in humans and mice – besides follicular B or B2 cells by extrafollicular reactions in a T-cell-independent mechanism (74–77). Although it has been less explored, some reports suggest that IgM-PCs could also come from follicular responses through GC reactions (78–80). Transitional B cells correspond to the most immature B cell type in the peripheral blood (74), while MZ B cells are B cells identified in the MZ of the spleen only (76). On the other hand, B1 B cells constitute a distinct B cell lineage, are part of the innate immune response, and generate effectors rapidly in the first stages of a humoral immune response (81). However, these possibilities have been barely explored during natural or experimental DENV infections.

As mentioned before, very few studies have focused on primary “natural” Abs during DENV infection, as an example, IgM can be found in secondary infections although, commonly, the average titers are lower than in primary ones (82). However, it has been reported that the human IgM response to DENV could be predominantly cross-reactive among DENV serotypes, and this IgM response is significantly higher in patients with SD than in patients with DF (acute phase) (83–85). Additionally, although it has been described that some IgM Abs have the ability to neutralize DENV, the epitopes that they are recognizing seem rather discontinuous or conformational (86).

Also, in the sera of DENV patients, it is possible to find IgM Abs cross-reacting with platelets, a finding which may contribute to explain in part DENV pathogenesis. Due to the polyclonal B cell activation that DENV would be causing, this could lead to IgM production (56, 83), but the cells producing these IgM Abs have not been assessed. In humanized BLT-NSG (NOD-*scid IL2r*γ*^*null*^* mice) immune-deficient mice, the Ab response was characterized by the lack of production of DENV-specific IgG but by the presence of DENV-specific IgM-secreting B cells, perhaps due to an elevated number of immature (transitional) B cells in the periphery (87). Research on primary “natural” Ab responses to DENV has been overlooked, and further studies are needed to elucidate their role during the infection, either in virus clearance or in pathogenesis. Despite the cross-reactivity of IgM Abs to DENV serotypes, apparently, they are not contributing to enhance the infection (88). However, they could be involved in the development of pathogenesis, as they can recognize self-Ags in platelets. It would seem that the enhancing properties of the Abs are restricted to the class-switched (IgG) memory ones.

Efforts to elucidate how is it that putative non-neutralizing memory Abs could enhance the disease have described *in vitro* using cell lines that through subneutralizing or non-neutralizing Abs, DENV infection suppresses innate cell immunity *via* FcγRs, thus facilitating viral replication (89–91). Similar experiments in human primary cell cultures seem to provide different pathways to enhance the infection by DENV *via* FcγRs (92, 93). Nonetheless, whether these mechanisms depend on particular FcγRs is still unclear (94, 95). Data from *in vitro* studies indicate that any specificity of monoclonal Abs to dengue might form infectious immune complexes (ICs), the major requirement being Ab concentration below that needed for neutralization (94, 96). To this respect, it has been described that the density of Ag-IgGs and FcγRs cross-linked on macrophages might influence phagocytosis and IL-10 production (97). Something similar could be happening in DENV infection.

## Long-Term (Memory) B Cell Responses and Memory Abs during DENV Infection

Regarding B cell biology, perhaps the most important function of B cells is to become Ab-producing cells. There are many good studies about Abs during DENV infection and recently about the DENV epitopes that induce them. However, the results have been somehow puzzling since neutralizing memory Abs make up a small fraction of the anti-DENV neutralization activity in human immune sera (4, 7, 8, 15, 98). Furthermore, certain Abs have been related to enhancing the infection in the mechanism called ADE, where memory Abs from a primary infection would be enhancing a secondary heterologous DENV infection (6). Only very recently were the first findings published over DENV epitopes that define the Ab responses in humans, as well as the mechanisms that the virus seems to use to infect host cells (7, 8, 15). Non-neutralizing memory Abs could render immature DENV particles infectious; thus, non-infectious viruses can enter *via* FcγRs and replicate efficiently, leading to more infected cells, potentially contributing to a more severe disease (23, 24).

Memory Abs, which are present before a secondary antigenic exposure occurs, constitute a very powerful evolutionary (anticipatory) strategy to cope with potential subsequent infections; this may allow the neutralization of a given pathogen before a second infection is well established (99). MBCs are the ones implicated in the Ag recall response and are rapidly activated during a secondary infection. MBC activation is faster, thus providing rapid protection against re-exposure to potentially dangerous Ags when MBCs differentiate to PCs and generate long-lasting B cell immunity (100). Released memory Abs by these PCs should neutralize DENV, as would be the case in homologous reinfections. However, during heterologous DENV reinfections, some LLPCs and MBCs might be apparently “responsible” for producing infection-facilitating Abs (101–103), an important issue that still needs to be well clarified. It has not been assessed in DENV infection how the affinity maturation develops during the first encounter with the virus and whether the affinity of MBCs is modified during a secondary heterologous infection.

Memory IgG Abs are generated mainly through T-cell-dependent reactions. Many of the non-neutralizing class-switched memory Abs during DENV infection are directed against epitopes found mainly on immature particles, such as the prM and E Ags (7, 8, 15, 27, 28, 104). These potentially enhancing-infection and non-neutralizing Ab responses are the dominant functional activities that are noted for the DENV-specific Abs encoded by MBCs, which predominate in the circulation even two or more decades following DENV infection (103). In addition, even neutralizing Abs may act, at least *in vitro*, as enhancers when at subneutralizing concentrations (7, 15, 102, 103). Furthermore, neutralizing class-switched memory Abs make up a small fraction of the anti-DENV binding and neutralization activity in human immune sera and are preferentially directed to EDIII (4, 7, 8, 15, 98). Interestingly, the most potent neutralizing DENV serotype-specific Abs bound to complex conformational epitopes found only on the intact viral particles (2, 5, 101, 102).

It has been reported that the B cell response detected early after primary DENV infection is predominantly serotype-specific, whereas responses detected early after secondary infection are predominantly serotype cross-reactive (56, 59). Abs detected during secondary infection recognize multiple serotypes of DENV E protein and have higher avidity to heterologous epitopes. Even after DENV infection, it is possible to observe in post-convalescent patients (6 months after primary infection) B cells reactive to heterologous E proteins at late time points, which were absent earlier (during the acute phase) (62). Additionally, PBs, which were generated from a very diverse, affinity-matured, and selected pool of MBCs and that did not proliferate extensively before differentiating into PBs, can also lead to the loss of dengue specificity because of this limited proliferation (63). Therefore, we might ask: is DENV inducing a preferential survival and/or generation of cross-reactive MBCs in order to ensure a successful infection during secondary encounters?

In the case of the last DENV vaccine (CYD-TDV), although few data are available on the generation of long-term immune memory, very recently, it was found in individuals after 5 years of vaccination, that DENV-specific MBCs are scarce in blood and secrete low amounts of Abs when stimulated. The circulating Abs showed low titers 5 years after vaccination, and these Abs from vaccinated individuals had limited *in vivo* efficacy against DENV2. Although the sample size was too small for definite conclusions, immune memory after vaccination with CYD-TDV appears relatively low (105).

## How are the DENV Ags Reaching the B Cell Follicles Inside the Regional (Draining) Lymph Nodes?

As mentioned before, cutaneously inoculated DENV does infect immune-competent mice and induces a strong GC response (50). On the other hand, long-lasting MBCs are generated through GCs (69), and DENV Ags inside B cell follicles are likely needed to drive these GC responses. However, it is not clear how DENV or DENV Ags are reaching first the draining lymph nodes (DLNs) regional to the inoculation site and then the B cell follicles inside these regional nodes. Indeed, it is still unclear, and under much recent scrutiny, how is it that many other Ags reach the follicles and initiate the activation of B cells. Some mechanisms have been described highlighting where and how follicular B cells encounter Ags. First is how Ags are reaching the DLNs; to this respect, it is known that Ag size is a major factor, e.g., particulate Ags (for instance, vesicular stomatitis virus, inert beads coated with Ag, etc.) and large ICs are bound by subcapsular sinus (SCS) macrophages. These macrophages act as sentinels to uptake incoming Ags and pathogens entering *via* the afferent lymph, and in some cases, they shuttle these Ags to underlying B cells (106–109). Murine macrophages of the LN SCS facilitate B cell activation *in vivo* by collecting and displaying native Ags (108, 110). By contrast, small Ags (under 70 kDa) are rapidly channeled into follicles *via* conduits. LN conduits constitute an effective fluid shunt between the SCS and the blood-vessel lumen where low-molecular-weight substances reach the lumen of high endothelial venules (111). It is proposed that either small Ags enter the follicles *via* small gaps in the floor of the LN sinus where they are bound by cognate B cells or that the majority of lymph-borne Ag enters the follicles through conduits first (107, 112, 113). Results have also demonstrated that cognate B cells could take up Ags directly from the conduits, possibly at the gaps between the fibroblastic reticular cells and the conduit, as identified by electron microscopy (107).

On the other hand, regarding the distribution of Ags inside the DLN, it is important if the encounter with that Ag is for the first time or if it is a second encounter with the same Ag. Studies on early Ag capture showed that while most Ags are trapped and degraded in the medullary region during their first encounter, in a subsequent challenge, Ag–Ab complexes are initially trapped in the floor of the SCS (114, 115). In both cases, small amounts of Ag manage to infiltrate the follicle and can be retained there on follicular dendritic cells (FDCs) for prolonged periods of time (115, 116). SCS macrophages capture and present intact Ag in the form of ICs, viruses, and virus-like particles, to activate follicular B cells (106, 108, 109). However, how is it that naive B cells are activated upon the first Ag encounter, and how they “decide” to go into the follicles to undergo GC reactions – or not – is still unclear for many Ags and completely unknown for DENV.

Moreover, classical studies have been very instructive describing the uptake of Ags by FDCs (116). FDCs are more prominent during GC reactions, and FDC retention of Ags is essential for clonal selection of B cells within GC (107). Whether Ag retention is also required for maintenance of DENV memory and effector B cells is not clear. Some studies support a role for Ag persistence in the maintenance of B cell memory (107, 117). However, how and whether DENV Ags are localized or persist on FDCs during primary or secondary infections is unknown. Are DENV Ags retained on FDCs after a primary DENV infection? And – if so – for how long? Do they participate in the affinity maturation and selection of B cells and in the generation of cross-reactive Abs during a secondary heterologous infection?

In the immune-deficient murine model (AG129 mice), it has been shown that macrophages from the SCS are important controlling the spreading of the DENV. These SCS macrophages contained NS1 protein, likely implying that they are trapping DENV Ags (118). Furthermore, we have shown the presence of E, prM, and NS3 DENV proteins inside B cell follicles and GCs, indicating that DENV Ags are indeed reaching DLNs and also raising the possibility that DENV might be even replicating in these lymphoid tissue compartments (50). However, how DENV Ags are finally reaching the LNs is not clear. We and others have found DENV-infected cutaneous dendritic cells (DC) in human cadaveric and non-cadaveric healthy skin explants infected *ex vivo* (119–121). DCs are specialized sentinel cells that uptake Ags at peripheral sites and travel to DLNs ferrying them to DC areas where B cells migrating toward the follicles are also likely to encounter these Ags (122). It is highly likely that DENV might be reaching DLNs also through cutaneous DCs or by the lymphatic circulation, but this needs to be formally demonstrated as almost nothing has been explored on this regard. Very recently, by flow cytometry, it was possible to find in intradermally DENV-infected immune-deficient mice [IFN-α/β receptor knockout (IFNAR) mice], migratory DENV-infected DCs in the skin-draining node. This suggests that dermal DCs may be carrying DENV and probably initiating the adaptive antiviral immune response (121). However, exactly where (and how) is DENV localized inside DLNs after cutaneous infection has not been evaluated. It is possible that DENV-infected DCs localize in the interfollicular zone or in the T-cell zone inside the DLNs, which would allow the activation of both follicular and extrafollicular B cells.

To explore how DENV (from the skin) might be reaching the DLNs, we did preliminary experiments tracking labeled DENV or ovalbumin (OVA, as a control) upon cutaneous inoculation. As reported by others (123, 124), OVA was found in the SCS of DLNs very early (1 h) after inoculation, but not DENV, which was only seen later in DLNs (unpublished data). Double labeling revealed that macrophages were apparently trapping DENV when it arrived into DLNs, either by lymph or in cells that might transfer the Ag to macrophages. Conceivable, skin-infected DCs might be ferrying DENV directly into DLNs (121). All this would suggest that skin-derived DENV enters DLN *via* lymph and that at rather early stages, incoming DENV seems contained by macrophages in the subcortical area, a phenomenon also described for other Ags such as *Salmonella adelaide* flagella (114).

## Concluding Remarks

Despite the fact that Ab responses are generated by B cells, we know surprisingly little about the (acute, chronic, or memory) B cell responses and the cellular mechanisms generating these Abs during DENV infections, both in humans and in animal models. It is assumed that B and T cells participate in both the protection and possibly in the putative enhancement of the disease. To know which epitopes are driving the different Ab responses and how is it that higher or lower Ab affinities are established during infection and whether these events influence protection or pathogenesis during the disease, could be very useful to design more efficient, and particularly in the case of dengue, safer vaccines. This might be especially relevant in the current situation of concomitant virus infections, such as Zika and Chikungunya, where the potential involvement of the immunological memory to DENV in the outcome of these infections needs to be clarified. Of note, it was described recently that the more advanced DENV vaccine (CYD-TDV) “is walking a tightrope” as the short-term safety profile was benign [the efficacy reported at the beginning of the clinical trials was 30.2% (125)], but upon 25 months of disease surveillance, it was difficult to withdraw definite conclusions (126, 127). Besides, prior immunity seems needed to the efficacy of this vaccine (125, 126). We need to understand better these findings before this vaccine can be declared safe. Thus, a major comprehension of the B cell responses in their different stages and compartments during natural DENV infections is critical to develop strategies to better counteract the upsurging, not only of DENV infections but also of other related emerging viral diseases.

## Author Contributions

Conceived the topics and designed the paper: JY-P and LF-R. Wrote and revised the paper: JY-P, LC-B, EA-M, RR-P, AE-G, and LF-R. Final approval of the version to be published: LF-R.

## Conflict of Interest Statement

The authors declare that the research was conducted in the absence of any commercial or financial relationships that could be construed as a potential conflict of interest.

## Abbreviations

ADE, antibody-dependent enhancement; DC, dendritic cell; DENV, dengue virus; DHF, dengue hemorrhagic fever; DLN, draining lymph node; DSS, dengue shock syndrome; E, envelope; EDI-III, envelope domain I-III; FcγR, Fc gamma receptor; FDC, follicular dendritic cell; GC, germinal center; IC, immune-complex; LLPC, long-lived plasma cell; M, membrane; MBC, memory B cells; MZ, marginal zone; NS, non-structural; OVA, ovalbumin; PB, plasmablast; PC, plasma cell; prM, precursor membrane; SCS, subcapsular sinus; SD, severe dengue.
